# Nutrition in the Bin: A Nutritional and Environmental Assessment of Food Wasted in the UK

**DOI:** 10.3389/fnut.2018.00019

**Published:** 2018-03-28

**Authors:** Karen A. Cooper, Tom E. Quested, Helene Lanctuit, Diane Zimmermann, Namy Espinoza-Orias, Anne Roulin

**Affiliations:** ^1^Nestlé Research Centre, Lausanne, Switzerland; ^2^WRAP, Banbury, United Kingdom

**Keywords:** food waste, sustainability, environmental impact, nutrition security, nutrient deficiencies, life cycle assessment

## Abstract

The UK currently has the most detailed, directly measured data for food wasted in the home. This includes information on the exact types of food wasted. These data allow calculation of the nutrients within that waste, as well as its environmental impact. The results progress the conversation beyond how much food is wasted or its energy content; it permits the implications for nutrition and sustainability to be assessed in detail. Data for UK household food waste were expressed as an average waste per capita for each type of food. Each food type was matched with an item (or group of items) from the UK Composition of Foods (7th Ed). The level of nutrients wasted was compared to UK Reference Nutrient Intakes (RNIs) for adult women (19–50 years, used as a proxy for general population requirements). The data were normalized into “nutrient days” wasted per capita per year, then into the number of complete diet days (for 21 nutrients plus energy). Results show that approximately 42 daily diets were discarded per capita per year. By individual nutrient, the highest losses were vitamin B_12_, vitamin C, and thiamin (160, 140, and 130 nutrient days/capita/year, respectively). For protein, dietary energy and carbohydrates, 88, 59, and 53 nutrient days/capita/year, respectively, were lost. Substantial losses were also found for under-consumed nutrients in the UK: calcium, which was mostly lost *via* bakery (27%) and dairy/eggs (27%). Food folate was mainly lost through fresh vegetables/salads (40%) and bakery (18%), as was dietary fiber (31 and 29%, respectively). Environmental impacts were distributed over the food groups, with wasted meat and fish the single largest contribution. For all environmental impacts studied, the largest contribution came from agricultural production. This paper shows that there are areas where interventions preventing food waste and promoting healthy eating could work together (e.g., encouraging consumption of vegetables or tackling overbuying, especially of unhealthy foods). Food manufacturers and retailers, alongside governments and NGOs, have a key role to minimize waste of environmentally impactful, nutrient-dense foods, for instance, by helping influence people’s behaviors with appropriate formulation of products, packaging, portioning, use of promotions, or public education.

## Introduction

Today, approximately a third of all food produced globally for human consumption is lost or wasted (1). This equals to approximately 1.3 billion tonnes per year. In the developing world, most of the losses occur in the early and middle parts of the supply chain due to lack of infrastructure for transportation, storage, cooling, and markets. In the developed world, a greater proportion of food is wasted in the retail part of the supply chain and during consumption (either in or out of the home) due to current commercial practices, lack of shopping planning, improper food handling in the home, confusion in the understanding of labeling date, or poor leftovers management (2, 3).

Food loss and waste data coming from the FAO (1) are expressed by weight (in tonnes). In 2013, the World Resource institute (WRI) converted these weights into kilocalories in order to demonstrate the global loss of nutritional energy (4). This illustrated differences that were not apparent before. For example, cereal losses accounted for 19% of total loss by weight but equaled 53% of the losses by kilocalories.

Today, 4,600 kcal/day of food are harvested for every person on the planet; of these, only around 2,000 kcal, on average, are eaten (5). If crops fed to livestock are included, European countries have approximately three times more food than required, while the USA has around four times more food than is needed.

Based on the above data, it has been reported that all the world’s 815 million hungry people (6) could be lifted out of energy/protein malnourishment on less than a quarter of the food that is wasted in the USA, UK, and Europe or by the circa 40 million tonnes of food wasted by USA households, retailers, and food services each year (7).

Many countries have generated data on the total quantity of food waste by sector. However, in the UK, WRAP—a not-for-profit organization working on sustainable use of resources—has also measured and reported on food waste from UK households to a high degree of detail, including a breakdown of the types of food thrown away. A recent example of this information was a report containing 2012 data (8). Even though a decrease in food waste had been observed between 2007 and 2012, further analysis indicated that the amount of edible food wasted at the household level represented about 15% of all food purchased (9): i.e., the food that could have been eaten but was discarded, usually, because it was not used in time, too much was prepared or served, or due to personal preference (8, 9). Importantly, for this paper, the high level of detail in these reports is sufficient to allow assessments of the amounts of nutrients and the environmental impacts associated with this waste.

Data on calorie loss lacks information on dietary quality, and so it is not possible to know specifically which macro- and micronutrients were lost. Using the full nutrient profile of foods in the state in which they are wasted can provide insight into where the nutrients are lost, especially if those nutrients match up to specific nutrient deficiencies in the population of interest. In the UK, these deficiencies are reported to be calcium, food folate, iron, vitamin D, and iodine with particular subgroups of the UK population (10, 11), as well as dietary fiber (12). This assessment also allows the calculation of the number of people that could be potentially fed with a balanced diet if less food was wasted. The paper also contains a detailed assessment of five environmental impact indicators: climate change, freshwater consumption scarcity, abiotic resource depletion, land use impacts on biodiversity, and impact on ecosphere/ecosystems quality.

This paper provides an approach to create a new level of information from existing food waste data. This new information can guide decisions on waste prevention and healthy eating: supporting the development of strategies for governments, as well as food processing and food retail companies to waste fewer nutrients and reduce their environmental impact. For instance, this information helps determine which items have the highest environmental impact and highest nutrient density. The results can also provide important information for public-facing campaigns relating to food waste and healthy eating.

## Materials and Methods

### Food Waste data

A WRAP report on the food and drink waste (referred to as food waste in this paper) generated in UK households formed the basis of this study (8). WRAP supplied detailed data on the weight of food waste by both food group (e.g., fresh fruit) and type (e.g., apples). The data cover food going to the following disposal routes: the general bin (usually going to landfill or incineration), council food waste collections (usually going to industrial composting or anaerobic digestion), down the sewer, home compost, fed to animals. Therefore, these are all included in the definition of food waste used within this paper.

The food waste data were measured by a range of methods. For food waste in the general bin and council food waste collections, information came from waste compositional analysis: i.e., sorting material found in these waste streams into food and non-food, and weighing each. The sample size for the waste compositional analysis was 1,799 households. For the food items, these were further categorized into different types of food. Information on food waste going to the sewer, home composted, or fed to animals was obtained using kitchen diaries: households recorded the types and amount of food going to each of these disposal routes for seven days in paper diaries. The main kitchen diary obtained data for a whole week from 948 households. More information can be found in the related WRAP report (13).

The data supplied were for 2012—the most recent year with detailed information on food group and type. There is a more recent estimate of total food waste in the UK for 2015 (14), and this shows that the amount wasted per capita in 2015 was similar to 2012. Therefore, it is likely that the analysis and results are still broadly relevant at the time of publication.

Previously, WRAP has reported food waste split by avoidable, possibly avoidable and unavoidable waste [see Ref. (8) for definitions]. However, in this paper, the food waste was reclassified into edible and inedible. Broadly speaking, the edible classification contains waste from the avoidable and possibly avoidable categories, and inedible contains unavoidable waste. The other change was that whole items containing edible and inedible fractions (e.g., a whole banana containing flesh and peel) were allocated into these two fractions (previously whole items thrown away were recorded as avoidable—no split was made). Splitting whole items into edible and inedible fractions was performed in accordance with Appendix B of the WRI Food Loss and Waste Standard (15).

For calculating nutritional waste, only the edible food waste was considered. It is recognized that some people may consume some items classified as inedible, e.g., apple cores; however, it was decided to use a definition similar to WRAP for avoidable: what most people in the UK would consider edible.

The report did not cover food waste at other stages in the supply chain, such as in agriculture, processing, retail, or food service; therefore, the figures generated represent at-home waste only.

### Population Size Data

As the food waste data were collected in 2012, the population of UK by mid-2012 (63.7 million) provided by the Office for National Statistics (16) was used to calculate the per capita waste at the population level.

### Modeling Nutritional Content

The UK Composition of Foods (Seventh Edition) was used for all nutrient content data of wasted food. For each item in the food waste list, an equivalent, or a group of equivalent items were selected as a nutritional proxy. This information is detailed in the supplemental information as an excel file. In some cases, the choice was not straightforward. For instance, when selecting a nutritional proxy for milk, described as a single amount covering fresh, UHT and goats’ milk, guidance was taken from the Family Food Purchase data for the year 2012 (17), which describes the breakdown of types of milk purchased annually. In this case, three proxies were chosen with the amount of wasted milk divided in ratio to amounts bought, i.e., full fat (19.7% total milk sold), skimmed (10.6% total milk sold), and semi-skimmed milk (69.7% total milk sold), with the assumption that the amounts wasted would mirror purchases. Where there was an option for fortified foods and not fortified foods, the unfortified option was most commonly selected. The exception to this would be breakfast cereals and milk powder, where a mixture was chosen as these are commonly fortified in the UK.

As a proxy for the overall population needs, the Dietary UK Reference Nutrient Intakes (RNIs) for moderately active women aged 19–50 were selected. For energy intake, 2,175 kcal was selected, which is the value for women aged 19–34 (18–20).

There is the potential for different researchers selecting the proxy foods to choose different options from those available. To minimize the impact of this effect, two nutrition scientists from different cultural backgrounds (UK and France) made the selections independently, and then differences were discussed with WRAP to agree on a final selection. This approach acted as a sensitivity test for the assessment, as the individual nutrients were calculated for both the individual and final set of foods, and the potential differences illustrated.

Classification of food items into groups is consistent with the system used in WRAP research and can be seen in the supplemental information (8). Vegetables and fruits are split according to culinary use rather than the botanical classification. These two groups are split into “fresh” and “processed” to differentiate between those purchased in a fresh or uncut state, and those purchased preserved or pre-prepared. The so-called “fresh” items include those which have been prepared at home, for example, potatoes that have been baked, boiled, or mashed, as it is assumed that the potatoes had been purchased raw (or home grown). The so-called “processed” items include those that were purchased dried, tinned, frozen, pickled, or otherwise processed.

### Calculating Nutrient Days

The amounts of nutrients were normalized by UK RNIs and by USA Recommended Daily Amounts (RDAs) as a sensitivity comparison (Table S1 in Supplementary Material). This resulted in a new metric, i.e., “nutrient days,” which can be defined as the total amount of the nutrient wasted per year per capita divided by the RNIs or RDAs. This is because each nutrient has a different unit (μg, mg, g) and also the relative importance of the nutrients are not necessarily related to their weight. As the lists differ also in the actual nutrients, the number of nutrients was limited to those where there was a UK RNI, though the USA RDAs for vitamins E and K were kept for some of the analyses for interest.

### Modeling Environmental Impact

The goal of this assessment was to carry out a comprehensive quantification of the environmental impacts attributed to the edible portion (as described above) of food waste generated in UK homes. This was possible due to the high level of disaggregation of data reported by WRAP in 2013 (8) and the larger availability nowadays of life cycle inventories for food ingredients, food processing, food storage, and food preparation at home. WRAP previously estimated some environmental impacts, namely greenhouse gas emissions and water footprint (8, 21). This paper includes a greater range of environmental metrics including abiotic resource depletion, impacts on ecosphere (ecosystem quality), land use biodiversity impacts, and freshwater consumption scarcity.

The functional unit chosen for this assessment was expressed as “the amount of food —averaged per capita and per day—disposed of as edible food waste in the average UK.” As family sizes vary, and households with more people waste more food (9), a per capita allocation was selected as a more equitable basis for calculation, consistent with the nutritional analysis. It would be then possible to infer the country level impacts of edible food waste.

The edible food waste consists of raw ingredients and processed foods (understood here as either manufactured or prepared at home) from each food category, as well as convenience food (ready to consume or requiring some heating process prior to consumption).

**Figure 1:**
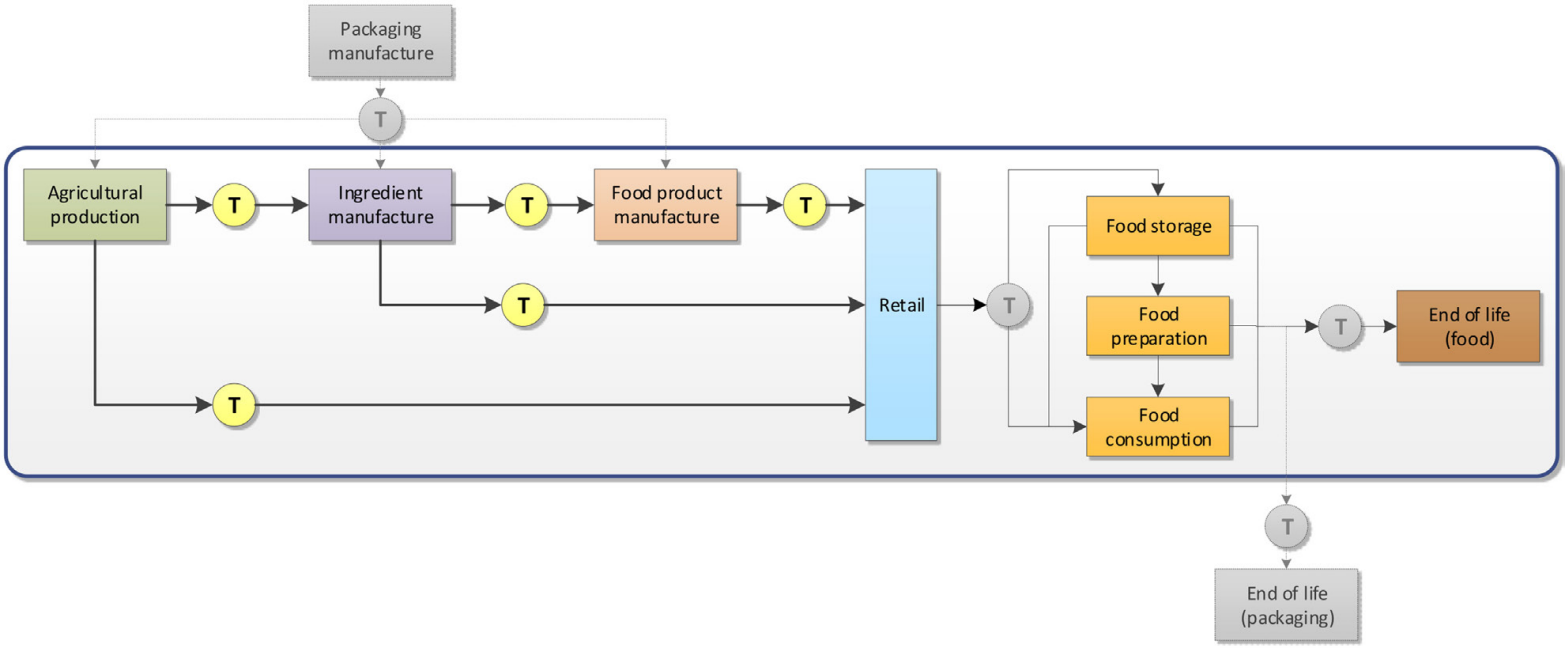
Scope of the assessment, covering life cycle stages from “cradle to grave.” Grayed and dashed elements are excluded from the system boundaries.

The scope of the assessment covered the life cycle stages from “cradle to grave” as shown in Figure 1.

Within the system boundaries, the following stages were considered:

(a) Agricultural production of food: cultivation of crops (perennial and annual) and animal husbandry;
(b) Manufacture of ingredients: includes, among others, slaughtering of animals, dairy processing, grain milling and processing, oil extraction and refining, and sugar production;
(c) Manufacture of food products: includes among others making bakery products and the manufacture of biscuits, confectionery, ice cream, beverages, and ambient, chilled, and frozen convenience food;
(d) Distribution and retail of the various food supplies (ingredients, processed food, convenience food) in accordance with their storage requirements (ambient, refrigerated, or frozen conditions) and shelf turnover (assumed here as 1, 3, or 5 days for high, medium, and low turnover rates, respectively);
(e) Refrigerated and frozen storage at home (assumed as 3 days storage in the refrigerator and 5 days in the freezer);
(f) Preparation of food at home: includes the use of electric appliances, such as heating up water using an electric kettle; frying, boiling, and steaming using an electric hob; oven preheating and baking at 220°C for a period of time (up to 30 min); additional ingredients such as vegetable oil for frying purposes, milk and water for the preparation of hot beverages, and water for boiling and steaming were also taken into account;
(g) End of life of edible food waste: landfilling for solid food and municipal waste water treatment for liquid food;
(h) Transportation of materials: considers the transport of materials from one stage to the next using different means of transportation (sea transport and road transport) and in accordance with the food requirements (ambient, chilled, or frozen transportation).

Packaging manufacture and its end of life were excluded from the assessment due to the uncertainty that would be introduced in the calculations by the large range of possible options for all the food items considered. Transportation of food from the point of purchase to the consumer homes and transportation of solid waste to the landfill were also excluded as they are deemed small contributors to the overall impacts. WRAP reports two additional (minor) end of life routes for food waste at home, namely home composting and animal feeding (8). These two routes were not considered in this assessment due to lack of reliable data for home composting conditions and the fact that it is not clear which food waste types would be fit for animal feeding purposes, nor which animal feed would be displaced by this use.

A key parameter identified in the assessment was the allocation of environmental impacts arising from the preparation of food at home between the portion of food actually consumed and the portion of food discarded as edible food waste. Using the data reported in the WRAP report (8), the following mass-based allocation factors were calculated:

– Food eaten: 81.5%
– Avoidable food waste: 11.1%
– Possibly avoidable food waste: 3.3%
– Unavoidable food waste: 4.1%.

As the benchmark value, the factor of 15% was chosen for the sum of avoidable and possibly avoidable food waste, i.e., equivalent to edible food waste. The rationale for this allocation step lies on the fact that energy and water were consumed with the purpose of preparing food, irrespective of it being consumed in its entirety or not. Moreover, the majority of food preparation processes at home are not necessarily efficient, in the sense that they cannot be scaled to the actual amount of food being prepared. For example, an electric oven has a fixed volume and requires preheating, whether it is used to roast a turkey for a family or to heat a ready-meal for one person.

Food supplies were generally considered regionally produced unless obviously otherwise, e.g., tropical fruits. In this case, it was assumed these were imported and the respective distance and means of transportation were included in the assessment.

A screening life cycle assessment was performed for each food item (a total of 424 items) reported in the WRAP report (8). The use of proxy data was minimal, due to adaptations made to available life cycle inventory data (regionalization to UK conditions and use of UK ingredients), as well as wider availability of data for agricultural commodities.

Background data (transportation, energy, water supply) and agricultural production data of raw ingredients were taken from publicly available databases such as ecoinvent v.3.3 (22), Agrifootprint v.2.0 (23), and Agribalyse v.1.3 (24). Additional data on raw materials and preparation of food at home were taken from the World Food LCA Database Project (25). Food manufacture and retail data were taken from Nestlé internal repositories.

The assessments were performed using the life cycle assessment software SimaPro v.8.3 (26). Five environmental impacts (at midpoint and endpoint levels), relevant and adequate to food systems, were evaluated in this study:

(a) Climate change (kg CO_2_-eq) (100 years) (27); this is a midpoint indicator measuring greenhouse gas emissions.
(b) Freshwater consumption scarcity (m3-eq) [AWaRE method—Available WAter Remaining, v.1.0 (28)]; this is a midpoint indicator characterizing the relative water available remaining in a watershed once the demands of ecosystems and humans have been met. The AWaRe method compares the relative availability of water in a watershed to the availability of water in a reference region, i.e., the global average. Availability of water in the calculation of this method is understood as water that is available in a watershed (water extracted from rivers, reservoirs, lakes, and aquifers) for other uses after the demand for water from aquatic ecosystems, humans, and human economic activities are met. For reference purposes, the world average value of water availability minus demand is 14 litres of water per month and per square meter, which is used as normalization factor in the AWaRe method.
(c) Land use impacts on biodiversity [PDF • m^2^ • year] [IMPACT World + /Land use method, v.0.05 (29)]; this is an endpoint indicator representing the changes in biodiversity (abundance and richness of species) due to different land uses.
(d) Abiotic resource depletion [kg Sb-eq] [CML 2001 method, v. 2.05 (30)]. This is a midpoint indicator; its characterization model combines “ultimate reserves” of non-renewable abiotic resources and their rates of extraction.
(e) Impacts on ecosphere/ecosystems quality [PDF • m^2^ • year] [Impact 2002 + method v. Q2.27 (31)]. This is an endpoint indicator. The method aggregates results on aquatic eutrophication, aquatic acidification, terrestrial acidification and nutrients, and ecotoxicity (aquatic and terrestrial).

## Results

### Nutrients and Complete Diets Wasted

The total weight of UK household food waste in 2012 was reported to be 7 million tonnes, which translates to 260 kg per household or 110 kg per capita per year (assuming average of 2.3 people per household). For edible food waste (approximately the sum of avoidable and possibly avoidable—see methods), this is estimated to be 5.4 million tonnes, 77% of the total waste or 85 kg per capita per year. As this paper presents information for *edible* food waste, the quantities differ somewhat from figures published by WRAP for *avoidable* food waste due to a slightly different definition.

**Figure 2:**
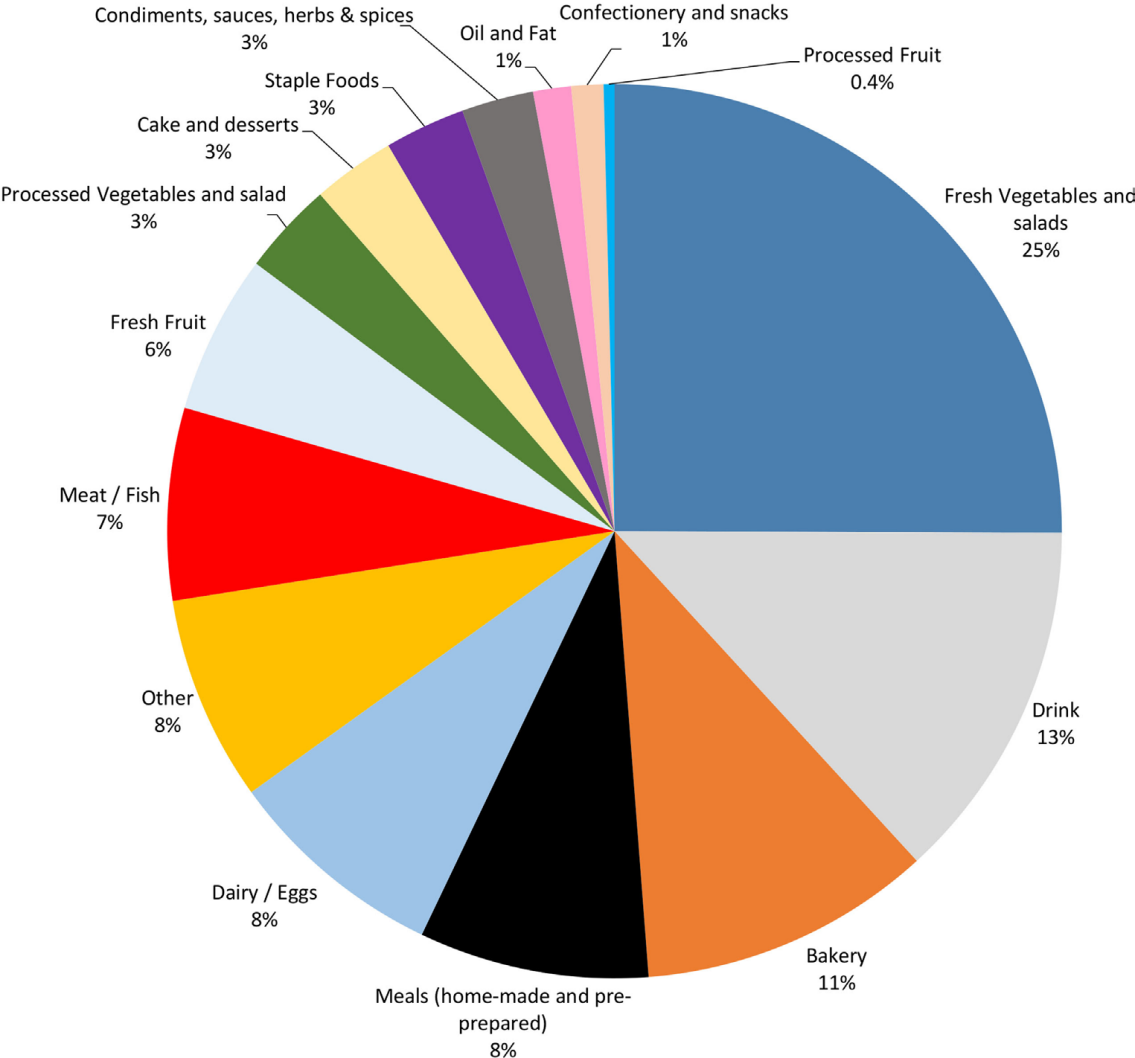
Edible household food waste by weight in the UK in 2012, separated by food category. WRAP data were classified as of time of calculation however, future alignment to the Food Loss and Waste Standard (15) is envisaged, which will likely create minor differences between edible and non-edible differentiation.

Household edible food waste spans every food category to varying degrees (Figure 2). The categories contributing the largest amount (by weight) to the total edible food waste are fresh vegetables and salads (25%), drink (13%), bakery (11%), dairy/eggs (8%), complete meals (8%), other foods (8%), meat/fish (7%), and fresh fruit (6%). The losses for the remainder of the food categories range between 0.4 and 3% by weight.

**Figure 3:**
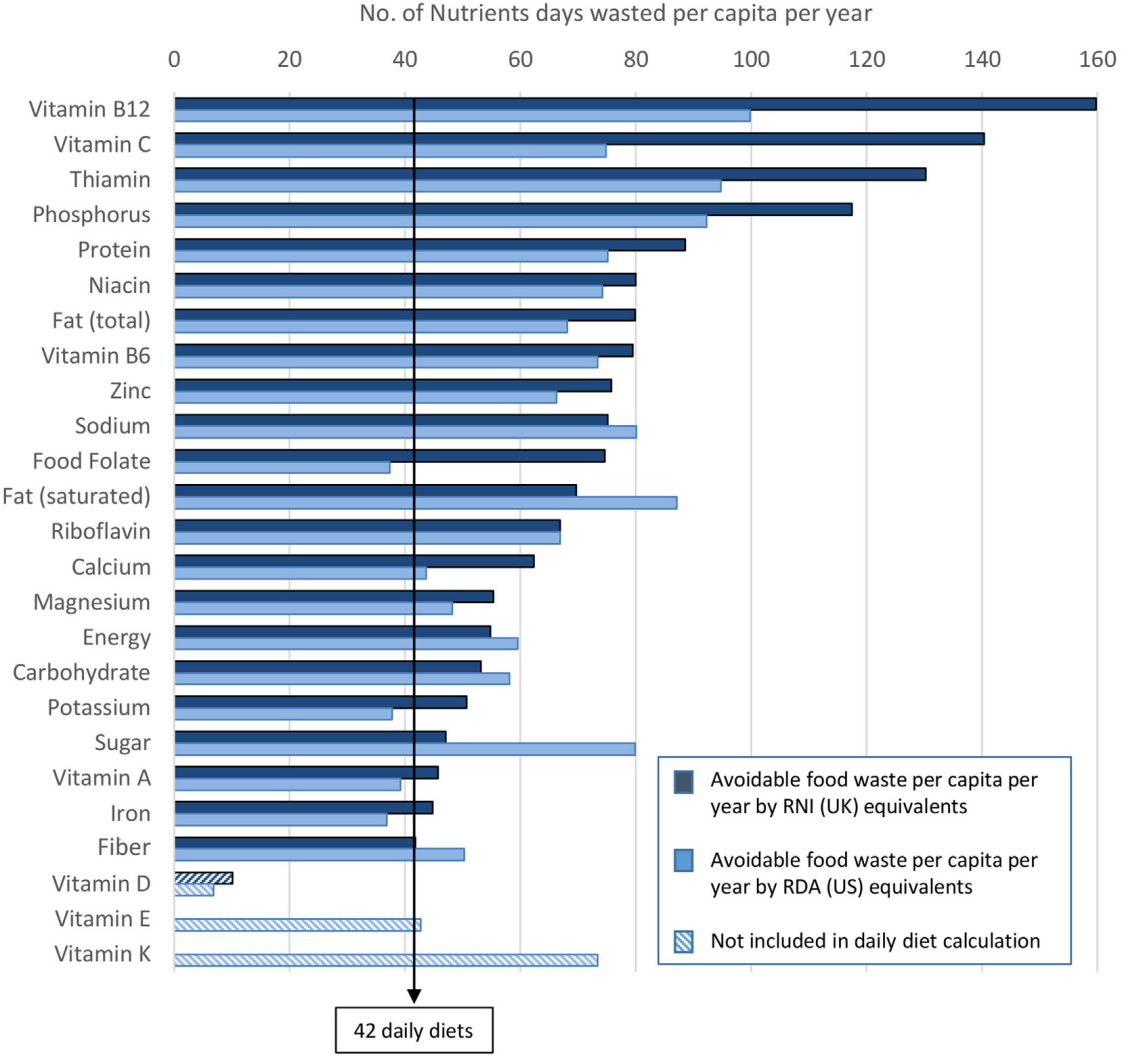
The amounts of individual nutrients wasted per capita per year in UK (in Nutrient days based either on UK-RNIs or on USA-RDAs). There is no bar for vitamins E and K for the UK as no RNI exist for these two vitamins. Results for vitamins D, E, and K not used for calculating the number of complete daily diets wasted (see text for more explanation). The arrow indicates the 42 daily diets, limited by fiber.

This analysis estimates that approximately 42 daily diets are contained within the average amount of edible household food waste thrown away per person each year (Figure 3). In other words, the typical food wasted by a person in the UK in a year would provide the nutrients required to meet the RNIs for 21 nutrients plus energy for 42 days (or 11% of a year). The limiting factor is fiber. For some nutrients, the number of days’ worth found in the waste is much higher. The top losses at single nutrient level are vitamin B_12_, where 160 nutrient days are wasted per capita per year, followed by vitamin C (140 nutrient days) and thiamin (130 nutrient days). This compares to 55 days’ worth of energy (or 15% of the recommended intake for a year). Vitamin D was incorporated later on in the analysis due to the new UK RNI (19). However, the full amount of vitamin D wasted is unlikely to be completely captured in this analysis as nutritional data for fortified foods were not usually selected to avoid inflating micronutrient levels (the exception being some dairy and breakfast cereal products where fortification is more common), and the fact that vitamin D is provided by exposure to sunlight (i.e., not only food sources). However, if vitamin D were included in the analysis, the number of daily diets wasted would be 10 (rather than 42).

If USA RDAs were used instead of UK RNIs, the pattern would be comparable, with some minor differences. Approximately 37 daily diets are thrown away per capita per year (compared to 42 with UK RNIs). 60 days’ worth of energy (rather than 55) is present in edible food waste. With vitamin D included in the analysis, approximately seven (rather than 10) complete daily diets are thrown away. In the USA, the top losses are vitamin B_12_ again (100 nutrient days), thiamin (95 nutrient days), and phosphorus (92 nutrient days).

**Figure 4:**
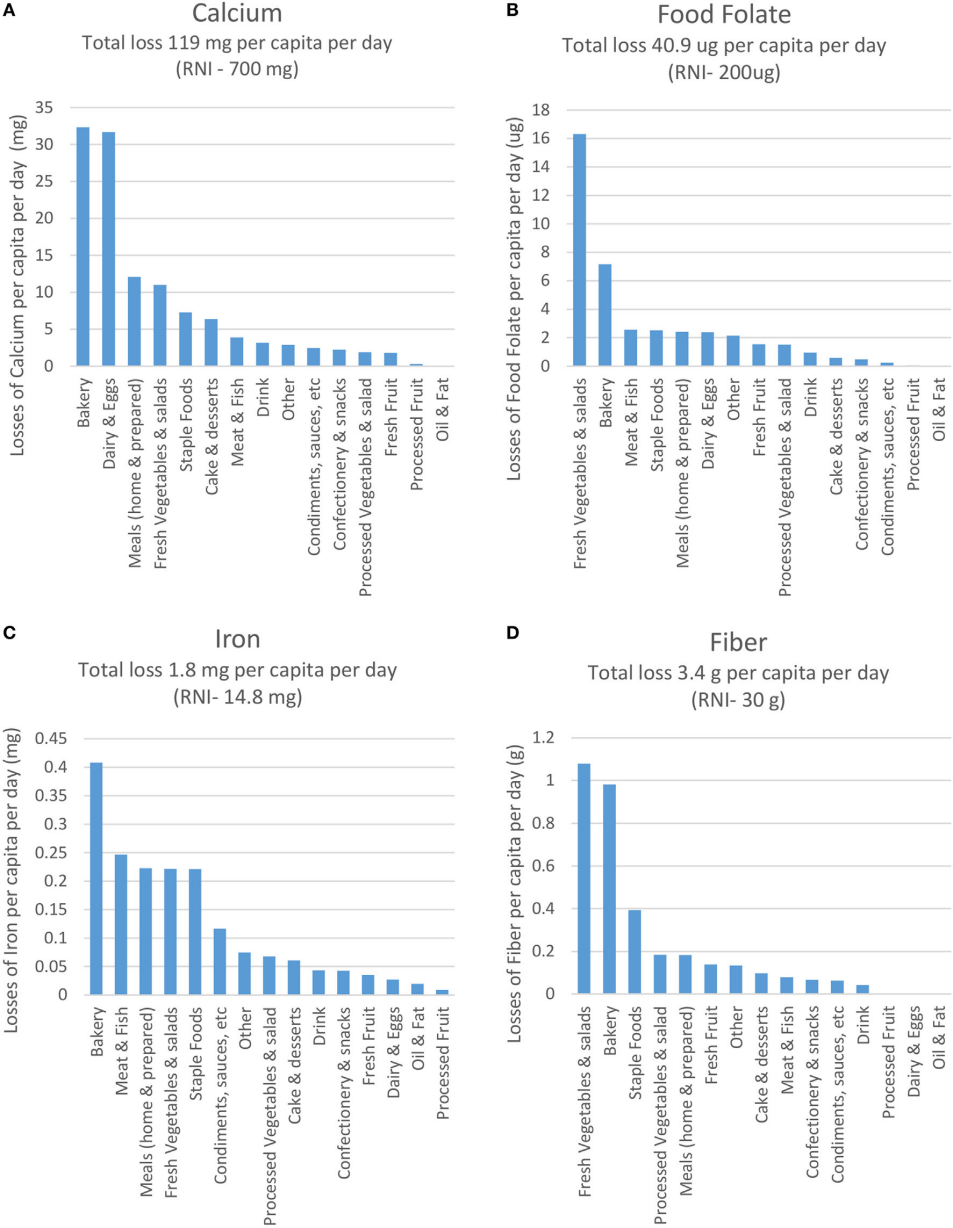
Amount of absolute wasted **(A)** calcium, **(B)** food folate, **(C)** iron, and **(D)** fiber, by food grouping per capita per day.

As each food was modeled for its nutrient content, it is possible to compare sources of foods wasted by individual nutrients. In the UK, recent dietary surveys indicate concern on micronutrients such as calcium, food folate (vitamin B9), iron, vitamin D, and iodine with particular subgroups of the UK population (10, 11), as well as dietary fiber (12). Data are shown in Figure 4 for key wasted foods containing calcium, food folate, iron, and fiber.

Results for calcium indicate that 119 mg/capita/day calcium is wasted, which is 28% of the RNI. The major sources (Figure 4A) are bakery (27% of the total) and dairy/eggs (27%). Within these food groups, it is estimated that the top losses come from semi-skimmed milk, bread (white, brown, and wholemeal), and hard cheese. For food folate, 40.9 μg/capita/day is wasted, representing 20% of the RNI. The main sources (Figure 4B) are fresh vegetables and salad (40%, often coming from leafy greens and potatoes) and bakery (18%). The total amount of iron wasted is 1.8 mg, which is 12% of the RNI. The major sources (Figure 4C) are from bakery (22%, e.g., bread), meat (14%, e.g., chicken, liver), and staples (12%, e.g., fortified breakfast cereals), as well as fresh vegetables and salad (12%, specifically potatoes). For dietary fiber, 3.4 g/capita/day are wasted, representing 11% of the RNI. The main contributing food categories (Figure 4D) are fresh vegetables and salad (31%, e.g., potatoes, carrots), bakery (29%, e.g., wholemeal and white bread), and staple foods (11%, e.g., bran-style breakfast cereals).

In summary, the typical food wasted by a person in the UK in a year would provide the nutrients required for 42 days (or 11% of the year). For some nutrients—notably vitamins B_12_ and C, and thiamin—the number of days’ worth found in the waste is much higher. For those nutrients where intakes are lower than recommendations:

– Bakery contributes substantially to the waste for calcium, folate, fiber, and iron
– Fresh vegetables and salad for food folate, dietary fiber, and iron
– Meat and fish for iron
– Dairy and eggs for calcium
– Staple foods for iron.

### Environmental Impact of Food Wasted

The greenhouse gas emissions associated with wasted edible household food is 0.9 kg CO_2_-equivalents per capita per day (Table 1). This would be roughly equivalent to 2.9 km traveled in a medium size car with a diesel engine (1.4–2 l engine). For a year, this totals 320 kg CO_2_ equivalents per capita, which in turn is equivalent to the impact of a round trip by car from Carlisle to the suburbs of London (1,060 km). For the whole of the UK, the total impact is 20.4 million tonnes CO_2_-eq per year, which would be equivalent to 6.5 million round trips across the United States by car (San Francisco to Maine, 10,400 km per round trip).

**Table 1:** Environmental impacts of edible food waste in the UK at a larger scale.

Functional unit (FU)	Climate change	Abiotic resource depletion	Impacts on ecosphere/ecosystem quality	Land use biodiversity impacts	Freshwater consumption scarcity
	(kg CO_2_-eq/FU)	(kg Sb-eq/FU)	(PDF × m^2^ × year/FU)	(PDF × m^2^ × year/FU)	(m^3^-eq/FU)
Per capita, per day	8.8E−01	3.2E−03	6.8E−02	7.0E−01	9.0E−01
Per capita, weekly	6.1E + 00	2.3E−02	4.8E−01	4.9E + 00	6.3E + 00
Per capita, annually	3.2E + 02	1.2E + 00	2.5E + 01	2.6E + 02	3.3E + 02
UK, annually	2.0E + 10	7.5E + 07	1.6E + 09	1.6E + 10	2.1E + 10

As for freshwater consumption scarcity, 0.9 m^3^-eq per capita per day are associated with wasted edible household food in the UK (Table 1). Therefore, the value of 0.9 m^3^-eq is slightly lower than the global average availability of water (defined as 1 m^3^-eq). This means that the freshwater consumption scarcity associated with food wasted per capita per day is almost equal to the current global average intensity of water demand. Note that the potential for deprivation of water for other uses in reality would be higher, since what is evaluated here is only the fraction of food that is not consumed, not accounting for the fraction of food that was actually consumed.

Non-renewable resource depletion associated with wasted edible household food in the UK adds to 3.2 × 10^−3^ kg Sb-eq per capita per day (Table 1). The main source of this loss is associated with the fuels used to generate electricity or for direct energy generation throughout the value chain. This is equivalent to the extraction of 160 g of crude oil per capita per day. At UK level, the total annual impact of 7.5 × 10^7^ kg Sb-eq is equivalent to the extraction of 2.7 × 10^4^ barrels of crude oil.

Wasted edible household food per capita per day is associated with a land use biodiversity impact of 0.7 PDF (potentially disappeared fraction) × m^2^ × year (Table 1). This impact is equivalent to what would be affected by the land occupation of 0.9 m^2^ per year by an annual arable crop. When considering the impact of the whole UK population in a year, the equivalent results are 1.6 × 10^10^ PDF × m^2^ × year, or the land occupation of 2.1 × 10^6^ ha of an annual arable crop.

Finally, the impacts on ecosystems quality associated with wasted edible household food in the UK are 6.8 × 10^−2^ PDF × m^2^ × year per capita per day (Table 1). This is an integrated environmental impact indicator combining ecotoxicity (aquatic and terrestrial), aquatic acidification, and aquatic eutrophication. For the purposes of explanation, if the results were expressed in terms of aquatic eutrophication only, then the impact would be equivalent to that caused by the emission of 6 g of phosphate ${\text{PO}}_{4}^{-}$to a water body. When looking at the impact at UK level, then the results add to 1.6 × 10^9^ PDF × m^2^ × year, which are equivalent to 1.4 × 10^5^ tonnes of phosphate emitted to water.

The environmental impacts associated with edible food waste produced by households in the UK are presented in Table 2 and in Table S2 in Supplementary Material (results per life cycle stage), and in Table S3 in Supplementary Material (results per food groups).

**Table 2:** Relative contribution per food group to overall nutritional content and environmental impact of edible UK household food wasted.

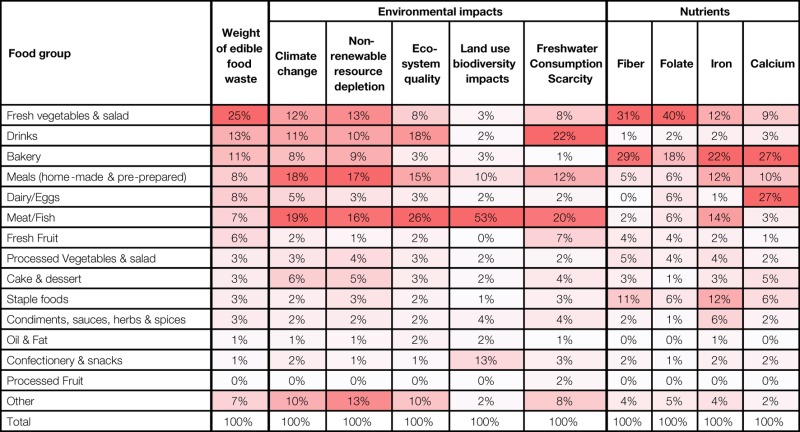

*Shading of cells indicates relative contribution of food group to each metric of impact*.

**Figure 5:**
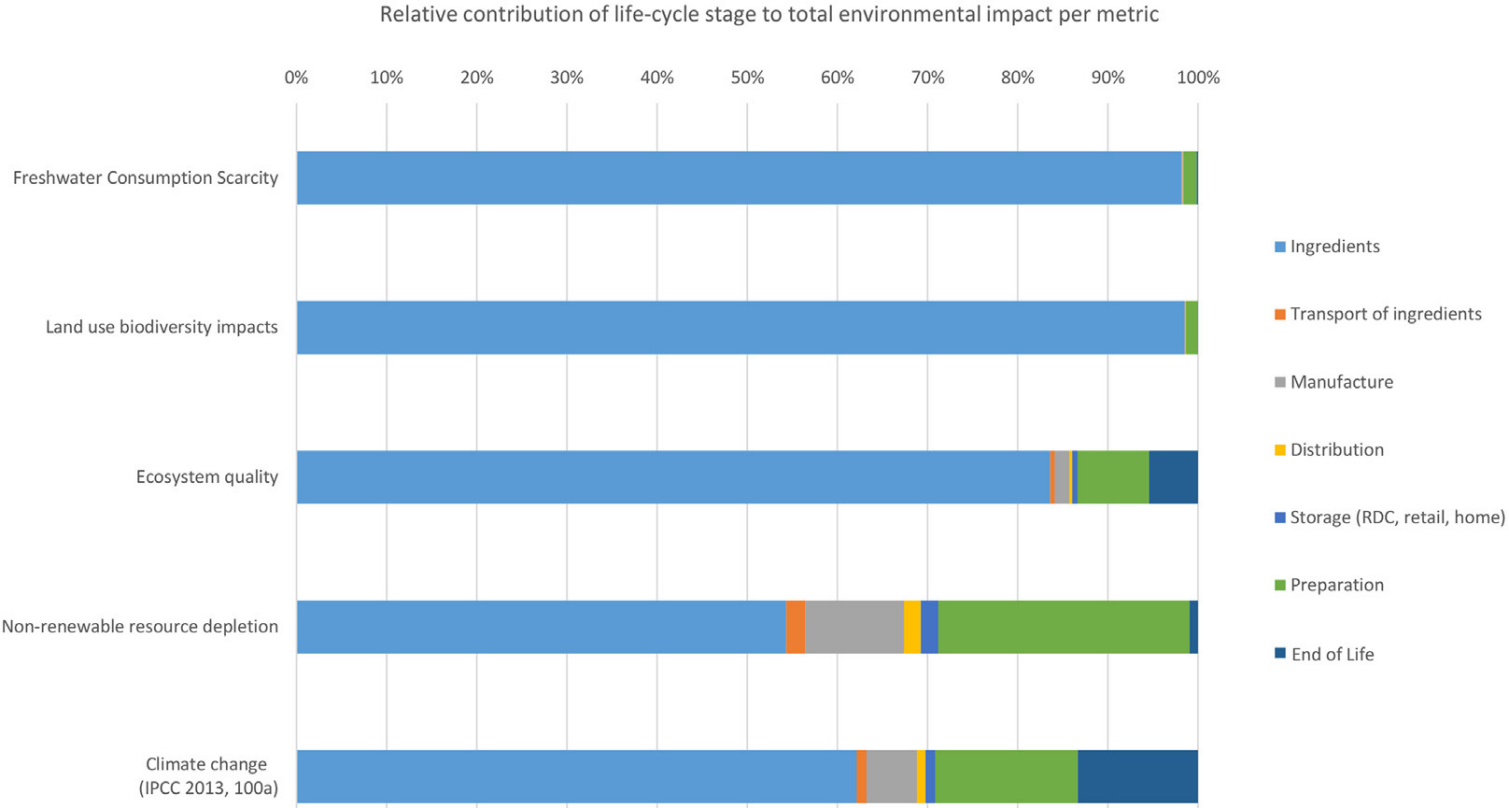
Relative contribution of the life-cycle stages to environmental impacts associated with UK household food waste.

In food life cycle assessments, the life cycle stages of agricultural production and the preparation of food typically contribute the most to the overall results. From Figure 5, it can be seen that the agricultural production of ingredients for food products contributes between 63% (for abiotic resource depletion) and 99% (land use biodiversity impacts) of the total impact for each indicator. This is explained by land use, land use change, agricultural practices, use of fertilizers (organic and synthetic), and irrigation for agricultural crops in geographical areas where water is scarce. Other notable contributions include the preparation of meals at home for climate change impacts (13%) and abiotic resource depletion (23%). This is largely due to the use in the assessed model of electric home appliances. The end of life of the food waste by landfilling contributes importantly to climate change (11%). Even though there is high uncertainty in the results for ecosystems quality, these can be explained by the contribution to eco-toxicity (soil, fresh water, and marine), eutrophication, and acidification attributed to agricultural practices (application of pesticides, herbicides, and fertilizers), the different ways to generate electricity in the UK (in particular, thermoelectric plants fueled by coal, natural gas, nuclear fuels), and the manufacture and end of life of household appliances (stove, oven).

When evaluating the results per food group, Table 2 shows that a small number of categories are contributing substantially to the total for each impact: for all environmental impacts, meat and fish, and meals are high contributors. Fresh vegetables and salad have a relatively high impact for climate change and non-renewable resource depletion due to the large amount of waste associated with this category; drinks contribute to ecosystem quality and freshwater consumption scarcity; confectionery and snacks contributes greatly to land use biodiversity impacts.

Some food groups have a disproportionately high environmental impact, given the amount of edible waste associated with them. For example, food groups containing food of animal origin (e.g., meat, fish, and meals) have substantially greater impacts associated with agricultural production than food groups of plant origin. This is explained largely by the fact that animal production systems have low conversion ratios (feed to final live weight). Furthermore, where food preparation entails the application of any type of heat treatment (re-heating by microwaving, boiling water, steaming vegetables, baking, roasting), the environmental impacts are larger. The contribution of drinks to freshwater consumption scarcity can be explained by the evident use of water either at manufacturing (e.g., bottled water or fizzy drinks) or at home to prepare a hot beverage (e.g., a cup of coffee or tea) and the water consumption embedded in the agricultural production of the ingredients used in a beverage, such as sugar, coffee, or milk.

The significance of the environmental impact of edible food waste is magnified when extrapolated to a larger scale, as shown in Table 1, where the results are presented per capita on a weekly and an annual basis, as well as for the whole of the UK in 2012 (population 63.7 million inhabitants).

### Overview of Wasted Nutrients and Environmental Impact

Bakery products, fresh vegetables and salad, staple food, dairy and eggs, and meat and fish were found to be the food groups contributing the most to the selected wasted nutrients. The environmental impact of those food groups and the wasted nutrients are summarized in Table 2. For each environmental impact and wasted nutrient, the table provides the percentage each food group contributes to the total. This provides a visual illustration of all the results in this paper, alongside the contribution to the weight of food waste.

For instance, wasted meat and fish is only 7% by weight of the total food wasted but contribute highly to all the environmental metrics (16–53%). In addition, they also contribute 14% of the iron found in the wasted food. However, they do not contribute greatly to wasted fiber, folate, or calcium. Conversely, bakery stands out as being a large contributor to the waste of all four nutrients of interest, but not strongly to any of the environmental impacts. Fresh vegetables and salad is the highest by weight, i.e., a quarter of total food wasted and represents large losses in key nutrients. Although this category is not the highest in terms of environmental impact (third in terms of climate change and non-renewable resource depletion), reducing these losses could contribute to meeting environmental targets. Where categories are less nutrient relevant but with a strong environmental impact, such as drinks, reducing these losses would contribute to environmental impact reduction, with minimal impact on beneficial nutrient provision.

This analysis highlights the highest food group contributors and could support awareness raising activities with science-based rationale. It could also help target the type of intervention activity, for instance, whether it is to encourage purchase and consumption (e.g., vegetables and salad) or to suggest reduced purchased amounts and, therefore, waste (e.g., drinks).

## Discussion

### Comparison With Other Approaches

This study takes publically available data and attempts to reshape the food waste discussion in terms of nutrition and environmental impact rather than by simply weight or calories. The reasoning behind this approach was to allow countries or organizations to focus their interventions on those foods that are most needed in the diet and also where wasting them contributes highly to the environmental impact of the food system. To the best of our knowledge, this is the first paper to make such an analysis on food waste combining nutritional and environmental impact.

Where a country has a known dietary deficiency, assuring access to foods rich in this nutrient (at affordable prices) is important. In the present analysis for the UK, known deficiencies could be matched to considerable losses of that nutrient *via* several different food groups. For instance, with the case of wasted iron, the food group contributing most is meat and fish, which is also the highest category in terms of climate change impact. This would give a clear indication that this food group is a likely potential focus for intervention on food waste in those communities or population segments where deficiencies have been identified.

A recent paper was published, which took a similar approach to converting food waste into nutrients and compared it with the dietary intake gaps within the US (32). The data source used was the USA’s Loss-Adjusted Food Availability data. This includes waste in retailers, hospitality and in the household, combining these three different “sectors.” Our paper differs in the fact that it focuses only on household waste and, therefore, can examine the impact just of households, drawing out specific conclusions without conflating a number of types of premises and sectors. The current paper also includes environmental impact. It further differs in terms of the data used for estimating food waste. The food waste data used by Spiker et al. (32) from the Loss-Adjusted Food Availability data infers the amount of waste by taking sales data (from a home panel) and comparing it to consumption data (using a recall method) where essentially the gap between the two is assumed to be wasted food. Any systematic uncertainty will have a disproportionately high impact on the estimates for food waste, as discussed by WRI in 2016 (15) *via* the guidance on mass balance. Recall methods for food consumption are known to create low estimates, especially for unhealthy foods. This has the effect that there appears to be relatively high levels of waste for instance for chocolate. The data used in the current paper comes from direct measurement of food waste, mainly *via* physical analysis of food in the household bins. Although there are uncertainties in these estimates [discussed at length by WRAP (13)], the fact that food waste was directly measured (rather than being inferred) is likely to lead to more accurate data.

In terms of findings, Spiker et al. (32) calculated that the losses in the USA in 2012 contained 1,217 kcal energy, 33 g protein, 5.9 g dietary fiber, 1.7 µg vitamin D, 286 mg calcium, and 880 mg potassium per capita per day. As our study covers household level food waste only, the expectation is that our figures would be considerably lower as it does not incorporate retailers or hospitality losses. Indeed, the current study calculated: 326 kcal energy, 10.9 g protein, 3.4 g fiber, 0.8 µg vitamin D, 120 mg calcium, and 486 mg potassium. The scale of the difference is quite large, especially for energy, and this could reflect the different methodologies mentioned above, the use of different food databases, and the fact that the studies focused on two different countries (potentially with different diets and waste-related behaviors).

Typically, research on the sustainability of different types of diets, either in the UK or in other countries, has focused on climate change as the representative environmental impact indicator. The present study has extended the analysis and calculated five different indicators for environmental impact, two of which were evaluated at high spatial resolution (land use impacts on biodiversity and freshwater consumption scarcity). A comparison of the results calculated for climate change here and in other published studies (see Table S4 in Supplementary Material) is not straightforward due to the different methods pursued to measure food waste but could provide, at least, some context. The impacts attributed to food waste as calculated in this study would represent between a third and a sixth of those attributed to food modeled as actually eaten by consumers following different diets (optimized, recommended, or self-reported but not necessarily comparable between them). For example, the environmental impact of food waste, as reported by Heller and Keoleian (33) in the US (1.4 kg CO_2_-eq per capita per day) (33) is around 40% higher than the value calculated in this study (0.88 kg CO_2_-eq per capita per day).

### Limitations

The first limitation of the study is that the waste data which forms the foundation of this analysis—despite being the most detailed of its type—is not perfect. There are a number of uncertainties associated with the estimates, which are well documented (34). Most significantly for this study, the uncertainties are relatively low for food types that are wasted most frequently.

Several assumptions were required in order to model the primary food waste data as nutrients lost: firstly, the selection of foods in the UK food composition database to represent each item in the food waste data. To minimize bias, two nutritionists from different cultural backgrounds (British and French) made the initial selection. Final agreements were made *via* guidance by WRAP, who collected the primary data. In general, more than one proxy item was selected in order to avoid skewing the nutrition results toward one food, which might be higher or lower on average in terms of nutrient density. The WRAP data provided 200 different groupings, with descriptors of what each group was intended to cover. This was converted into 424 items from the UK Composition of Food database. Some proxy items may be duplicated for food groupings where it was warranted, such as bakery groups for standard bread and other bakery products.

The UK food database does not include many fortified foodstuffs compared to the USDA food database, reflecting the fact that fortified foods are more common in the USA. However, it could mean that some nutrient losses are unaccounted for if UK households are purchasing fortified foods and wasting them.

Our study also does not include directly degradation losses in nutrient content at different parts of the food chain through storage losses, food preparation, etc. However, the nutrition level taken for each of the foodstuffs relates to the measurement taken at the point of waste, so error due to uncaptured losses is likely to be minimal. This is a strength of the study as it captures the nutrient content of cooked losses as well as raw losses.

In order to make a meaningful comparison between losses of nutrients, rather than food groups, the data were normalized by using UK-RNIs, resulting in the data units being number of RNIs or number of Nutrient days, as well as USA RDAs. The use of RNIs means that the calculation of the number of complete diets wasted is conservative. RNIs are deliberately set at a high level to ensure the number incorporates the needs of 97.5% of the population. If Estimated Average Requirements had been used, which represents the requirements of 50% of the population, the number of nutrient days would have been considerably higher. The selection of a female adult to represent the population requirements is also a limitation. For instance, the requirements for children are smaller, and so, if calculated at this level, the number of complete diets would be higher to satisfy child needs. Conversely, for some nutrients, adult males require more, and so this would be an under-representation.

### Future Studies

Future studies could take into account waste in places other than the household to give a more complete picture. Food consumption out of home is of particular interest since a substantial minority of food is eaten away from the home in the UK. The study could also be replicated for other countries, which compile waste data of good quality. It would also be interesting to simplify the method used here by looking for single proxies for food subgroups that deliver a close enough result, for both nutrition and environmental impact, so that useful data which can guide interventions is generated more easily. This would increase the feasibility of this method being implemented in countries lacking detailed food waste data.

### Providing Guidance for Interventions

Many organizations are implementing a range of interventions to reduce food waste, with many focusing on food wasted in the home within developed countries. Existing initiatives include consumer-facing campaigns focused both on attitudes toward food waste and practical tips to support change, and changes by food processors, manufacturers, and retailers to the way products are formulated, packaged, and sold to make it easier for the public to buy the right amount for their needs and use up what they buy. Such an approach to calculating food loss and waste can also be used by industry to help them focus their efforts on reductions across the value chain, i.e., loss of key ingredients upstream or product waste downstream.

One particular issue is the degree to which messages and campaigns on food waste and healthy eating could be combined into a single campaign, streamlining communication with the public—saving money and hopefully leading to greater change. This paper helps explore this issue. The results indicate that there are certain food types—vegetables being the most notable—that are wasted in relatively high amounts, contain many key nutrients (with positive health outcomes) and are not, on average, eaten in sufficient quantity by the UK population. In the case of vegetables, these nutrients include fiber, food folate, and iron. In such cases, our results demonstrate there is a clear and mutual benefit to initiatives and interventions that help people consume the vegetables they buy: it leads to less waste, reduced environmental impact, and generally better diet.

As an example of how the results in this paper could be used in a public-facing campaign, many organizations focus on preventing types of food relevant to them. For example, trade bodies representing meat production may want to understand the issues relating to meat disposed of from the home, to form the basis of an information campaign. Table 2 allows them to see—at a glance—the nutritional and environmental factors that are notable for that food group. This allows them to tailor a campaign to these particular issues—focusing a meat campaign on environmental impacts or a fruit campaign on wasted nutrients. In a similar way, a campaign being developed on, say, the water consumption associated with food waste can use Table 2 to determine the most important foods to illustrate this issue (in this case, drinks, and meat and fish).

With foods that are not associated with such positive health outcomes (e.g., sugar-rich foods), there is a potential conflict between initiatives aimed at reducing waste (e.g., a simplistic approach that asks people to clear their plates) and improving diets. In such cases, initiatives aimed at helping people to prepare and serve an appropriate amount of food become important, as noted by Neff et al. (35). In addition, appropriate pack sizes (36) and plate sizes (37) can both potentially have a positive impact on waste and health. Where reduced amounts of food waste are not accompanied by increased consumption, then this will reduce demand with lower environmental impacts for food.

Another area where there is the potential to make a positive difference to both health and waste is addressing over-purchasing, especially of unhealthy foods (35). Food purchased beyond the needs of a household usually ends up being thrown away or eaten as part of excess consumption. A recent report (38) suggests that multi-buy offers (e.g., three for the price of two) and high levels of discount are both associated with over-purchasing: i.e., extra purchases from these types of offers are only partially compensated by reduced purchases of either other items or future purchases. In the UK, retailers are in a strong position to influence the type of discounts used to minimize over-purchasing and could be instrumental in bringing about positive change.

## Conclusion

Approximately 42 complete daily diets are discarded per capita per year in the UK. The climate change impact of this waste is 320 kg CO_2_ equivalents per capita per year, which is equivalent to the impact of a round trip by car from Glasgow to London (1,060 km). By combining environmental and nutritional assessment of food waste, it is feasible to identify those wasted foods where the impact is particularly high both on the environment and on the diet. This can assist with targeting communication-based interventions—on food waste, diet, or a combination of the two. It can also help trigger innovation from retailers and food companies, for instance to supply recipes, new product ideas for highly wasted foods, with adapted portioning, packaging, and pricing.

## Author Contributions

AR, KC, and HL conceived the study. KC and DZ made the nutritional assessment of the food waste data, NE-O made the environmental assessment of the food waste data. HL wrote the introduction and contributed to the combined data analysis. TQ contributed to the combined data analysis and presentation of data. All authors contributed to the writing of the manuscript and revisions.

## Conflict of Interest Statement

KC, DZ, HL and NE-O are all employed by Nestlé. AR is recently retired from Nestlé. TQ declares that the research was conducted in the absence of any commercial or financial relationships that could be construed as a potential conflict of interest.
