# Supplementary material for: Nutrition in the Bin: A Nutritional and Environmental Assessment of Food Wasted in the UK
Source: Front Nutr. 2018 Mar 28;5:19. doi: 10.3389/fnut.2018.00019 (PMC5882835; doi:10.3389/fnut.2018.00019)
Supplement: Supplementary file 1 [file table_1.docx]

# Supplementary Material

**SM 1.** A comparison between selected current UK’s Reference Nutrient Intakes for women (19-34 years for energy and 19-50 years for micronutrients) and USA RDAs for women aged 19-30 years.

| **Nutrient** | **UK RNIs** | **USA RDAs** | **Unit** | **Difference** |
| --- | --- | --- | --- | --- |
| Calcium | 700 | 1000 | mg/d | Lower UK RNI |
| Carbohydrate | 260 | 238 (130) | g/d | Higher UK RNI |
| Energy | 2175 | 2000 | kcal/d | Higher UK RNI |
| Fat (saturated) | 20 | 16 | g/d | Higher UK RNI |
| Fat (total) | 70 | 82 | g/d | Lower UK RNI |
| Fiber | 30 | 25 | g/d | Higher UK RNI |
| Food Folate | 200 | 400 | µg/d | Lower UK RNI |
| Iron | 14.8 | 18 | mg/d | Lower UK RNI |
| Magnesium | 270 | 310 | mg/d | Lower UK RNI |
| Niacin | 13 | 14 | mg/d | Lower UK RNI |
| Phosphorus | 550 | 700 | mg/d | Lower UK RNI |
| Potassium | 3500 | 4700 | mg/d | Lower UK RNI |
| Protein | 50 | 53 (46) | g/d | Lower UK RNI |
| Riboflavin | 1.1 | 1.1 | mg/d | None |
| Sodium | 1600 | 1500 | mg/d | Higher UK RNI |
| Sugar | 90 | 53 | g/d | Higher UK RNI |
| Thiamin | 0.8 | 1.1 | mg/d | Lower UK RNI |
| Vitamin A | 600 | 700 | µg/d | Lower UK RNI |
| Vitamin B12 | 1.5 | 2.4 | µg/d | Lower UK RNI |
| Vitamin B6 | 1.2 | 1.3 | mg/d | Lower UK RNI |
| Vitamin C | 40 | 75 | mg/d | Lower UK RNI |
| Vitamin D | 10 | 15 | µg/d | Higher UK RNI |
| Vitamin E |  | 15 | mg/d | No UK RNI |
| Vitamin K |  | 90 | µg/d | No UK RNI |
| Zinc | 7 | 8 | mg/d | Lower UK RNI |
