# Supplementary material for: Nutrition in the Bin: A Nutritional and Environmental Assessment of Food Wasted in the UK
Source: Front Nutr. 2018 Mar 28;5:19. doi: 10.3389/fnut.2018.00019 (PMC5882835; doi:10.3389/fnut.2018.00019)
Supplement: Supplementary file 2 [file table_2.docx]

# Supplementary Material

**SM 2.** Life cycle impact assessment of edible food waste by households in the UK. Results shown per life cycle stage.

| **Life cycle stage** | **Climate change** | | **Abiotic resource depletion** | | **Impacts on ecosphere / Ecosystem quality** | | **Land use biodiversity impacts** | | **Freshwater Consumption Scarcity** | |
| --- | --- | --- | --- | --- | --- | --- | --- | --- | --- | --- |
|  | [kg CO_2_-eq/capita*day] | [%] | [kg Sb-eq/capita* day] | [%] | [PDF*m^2^* year/ capita*day] | [%] | [PDF*m^2^* year/capita* day] | [%] | [m^3^-eq / capita* day] | [%] |
| Ingredients | 5.5E-01 | 62.1% | 1.7E-03 | 54.3% | 5.7E-02 | 83.6% | 6.9E-01 | 98.5% | 8.8E-01 | 98.1% |
| Transport of ingredients | 9.5E-03 | 1.1% | 6.8E-05 | 2.1% | 3.4E-04 | 0.5% | 2.2E-04 | 0.0% | 4.7E-04 | 0.1% |
| Manufacture | 4.9E-02 | 5.6% | 3.5E-04 | 10.9% | 1.1E-03 | 1.6% | 6.7E-04 | 0.1% | 9.7E-04 | 0.1% |
| Distribution | 8.4E-03 | 1.0% | 6.1E-05 | 1.9% | 2.4E-04 | 0.4% | 2.2E-04 | 0.0% | 4.3E-04 | 0.0% |
| Storage (retail, home) | 9.3E-03 | 1.1% | 6.2E-05 | 1.9% | 3.9E-04 | 0.6% | 1.8E-04 | 0.0% | 6.7E-04 | 0.1% |
| Preparation | 1.4E-01 | 15.8% | 9.0E-04 | 27.9% | 5.4E-03 | 8.0% | 9.0E-03 | 1.3% | 1.3E-02 | 1.4% |
| End of life | 1.2E-01 | 13.4% | 3.0E-05 | 0.9% | 3.7E-03 | 5.4% | 3.0E-04 | 0.0% | 1.3E-03 | 0.1% |
| **TOTAL** | **8.8E-01** |  | **3.2E-03** |  | **6.8E-02** |  | **7.0E-01** |  | **9.0E-01** |  |
