# Supplementary material for: Nutrition in the Bin: A Nutritional and Environmental Assessment of Food Wasted in the UK
Source: Front Nutr. 2018 Mar 28;5:19. doi: 10.3389/fnut.2018.00019 (PMC5882835; doi:10.3389/fnut.2018.00019)
Supplement: Supplementary file 3 [file table_3.docx]

# Supplementary Material

**SM 3.** Life cycle impact assessment of edible food waste by households in the UK. Results shown per food group and substantiating results in Table 2 of the main manuscript.

| Food group | Climate change | Abiotic resource depletion | Impacts on ecosphere / Ecosystem quality | Land use biodiversity impacts | Freshwater Consumption Scarcity |
| --- | --- | --- | --- | --- | --- |
|  | [kg CO_2_-eq/  capita*day] | [kg Sb-eq/  capita*day] | [PDF*m^2^*year/  capita*day] | [PDF*m^2^*year/  capita*day] | [m^3^-eq / capita*day] |
| Bakery | 6.6E-02 | 3.0E-04 | 2.3E-03 | 2.4E-02 | 1.2E-02 |
| Cake & dessert | 5.0E-02 | 1.7E-04 | 2.2E-03 | 1.7E-02 | 4.0E-02 |
| Condiments, sauces, herbs & spices | 1.4E-02 | 7.4E-05 | 1.1E-03 | 2.7E-02 | 3.7E-02 |
| Confectionery & snacks | 1.6E-02 | 4.4E-05 | 7.9E-04 | 9.0E-02 | 2.9E-02 |
| Dairy / eggs | 4.4E-02 | 1.0E-04 | 2.0E-03 | 1.4E-02 | 2.1E-02 |
| Drinks | 9.3E-02 | 3.2E-04 | 1.3E-02 | 1.7E-02 | 1.9E-01 |
| Fresh fruit | 1.4E-02 | 3.8E-05 | 1.4E-03 | 3.7E-04 | 6.0E-02 |
| Fresh vegetables & salad | 1.1E-01 | 4.2E-04 | 5.7E-03 | 1.9E-02 | 7.3E-02 |
| Meals (home-made and pre-prepared) | 1.6E-01 | 5.6E-04 | 1.0E-02 | 7.1E-02 | 1.1E-01 |
| Meat / fish | 1.7E-01 | 5.3E-04 | 1.8E-02 | 3.7E-01 | 1.8E-01 |
| Oil & fat | 9.3E-03 | 2.7E-05 | 1.5E-03 | 1.4E-02 | 1.1E-02 |
| Other | 8.5E-02 | 4.1E-04 | 7.1E-03 | 1.6E-02 | 7.2E-02 |
| Processed fruit | 2.8E-03 | 1.4E-05 | 1.4E-04 | 3.9E-04 | 1.5E-02 |
| Processed vegetables & salad | 2.8E-02 | 1.4E-04 | 1.8E-03 | 1.3E-02 | 1.7E-02 |
| Staple foods | 2.0E-02 | 8.6E-05 | 1.2E-03 | 1.0E-02 | 2.7E-02 |
| **TOTAL**   \| Bakery \| 6.6E-02 \| 3.0E-04 \| 2.3E-03 \| 2.4E-02 \| 1.2E-02 \| \| --- \| --- \| --- \| --- \| --- \| --- \| \| Cake & dessert \| 5.0E-02 \| 1.7E-04 \| 2.2E-03 \| 1.7E-02 \| 4.0E-02 \| \| Condiments, sauces, herbs & spices \| 1.4E-02 \| 7.4E-05 \| 1.1E-03 \| 2.7E-02 \| 3.7E-02 \| \| Confectionery & snacks \| 1.6E-02 \| 4.4E-05 \| 7.9E-04 \| 9.0E-02 \| 2.9E-02 \| \| Dairy / eggs \| 4.4E-02 \| 1.0E-04 \| 2.0E-03 \| 1.4E-02 \| 2.1E-02 \| \| Drinks \| 9.3E-02 \| 3.2E-04 \| 1.3E-02 \| 1.7E-02 \| 1.9E-01 \| \| Fresh fruit \| 1.4E-02 \| 3.8E-05 \| 1.4E-03 \| 3.7E-04 \| 6.0E-02 \| \| Fresh vegetables & salad \| 1.1E-01 \| 4.2E-04 \| 5.7E-03 \| 1.9E-02 \| 7.3E-02 \| \| Meals (home-made and pre-prepared) \| 1.6E-01 \| 5.6E-04 \| 1.0E-02 \| 7.1E-02 \| 1.1E-01 \| \| Meat / fish \| 1.7E-01 \| 5.3E-04 \| 1.8E-02 \| 3.7E-01 \| 1.8E-01 \| \| Oil & fat \| 9.3E-03 \| 2.7E-05 \| 1.5E-03 \| 1.4E-02 \| 1.1E-02 \| \| Other \| 8.5E-02 \| 4.1E-04 \| 7.1E-03 \| 1.6E-02 \| 7.2E-02 \| \| Processed fruit \| 2.8E-03 \| 1.4E-05 \| 1.4E-04 \| 3.9E-04 \| 1.5E-02 \| \| Processed vegetables & salad \| 2.8E-02 \| 1.4E-04 \| 1.8E-03 \| 1.3E-02 \| 1.7E-02 \| \| Staple foods \| 2.0E-02 \| 8.6E-05 \| 1.2E-03 \| 1.0E-02 \| 2.7E-02 \| \|  \| **8.8E-01** \| **3.2E-03** \| **6.8E-02** \| **7.0E-01** \| **9.0E-01** \| | **8.8E-01** | **3.2E-03** | **6.8E-02** | **7.0E-01** | **9.0E-01** |
