# Supplementary material for: Nutrition in the Bin: A Nutritional and Environmental Assessment of Food Wasted in the UK
Source: Front Nutr. 2018 Mar 28;5:19. doi: 10.3389/fnut.2018.00019 (PMC5882835; doi:10.3389/fnut.2018.00019)
Supplement: Supplementary file 4 [file table_4.docx]

# Supplementary Material

**SM 4.** Comparison of dietary climate change impact.

| Reference | Functional unit | Climate change impact  [kg CO_2_-eq/FU] | Comments |
| --- | --- | --- | --- |
| This study | 227.6 g edible food waste per capita per day | 0.88 | Environmental impact of edible food waste. |
| Wilson *et al.* (2013) (39) | 1204 g food consumed per capita per day | 5.25 | Optimized diet for New Zealand conditions, achieving all nutrients for minimum cost. Main meal is based on minced beef, plus standard breakfast and lunch. Beverages and water not explicitly included. |
| Masset *et al.* (2014) (40) | 2922 g food consumed per capita per day (men) | 4.691 | Average male French diet, including beverages. |
| Masset *et al.* (2014) (40) | 2813 g food consumed per capita per day | 3.797 | More sustainable French diet, including beverages. |
| Macdiarmid *et al.* (2012) (41) | 1566 g food consumed per capita per day | 2.43 | UK Dietary requirements for an adult woman (19 – 50 y old); diet optimized to achieve low GHG emissions. Scope of the assessment up to the regional distribution center only. |
| Scarborough *et al.* (2014) (42) | 2000 kcal per capita per day | 5.63 | Medium meat eaters (50 – 99 g day) in the UK. |
| *Bälter et al*. (2017) (43) | Daily average diet | 4.7 | Self-reported diets in Sweden, as part of the LifeGene study |
| Heller and Keoleian (2014) (33) | 2000 kcal per capita per day | 5.0 | Recommended diet for US. This assessment includes results for food consumed (3.6 kg CO_2_-eq) and food waste (1.4 kg CO_2_-eq) |
